# Health technology assessment for digital health Technologies in India: a framework for action

**DOI:** 10.1017/S0266462324000345

**Published:** 2024-11-04

**Authors:** Denny John, Abhirup Dutta Majumdar, Rakesh N. Pillai, Sajda Khatoon, Paramita Bhattacharya, Nirmalya Mukherjee, Jaideep C. Menon, K. Pavithran, Anand Kumar, Mihajlo Jakovljevic

**Affiliations:** 1 Faculty of Life and Allied Health Sciences, Ramaiah University of Applied Sciences, Bengaluru, India; 2 Independent Statistician, Kolkata, India; 3 Epifractals Pvt. Ltd., Trivandrum, Kerala, India; 4 Research Officer, Centre for Public Health Research, MANT, Kolkata, India; 5 Centre for Public Health Research, MANT, Kolkata, India; 6 Department of Preventive Cardiology, Amrita Institute of Medical Sciences & Research, Kochi, India; 7 Department of Medical Oncology, Amrita Institute of Medical Sciences & Research, Kochi, India; 8 Department of Neurology, Amrita Institute of Medical Sciences & Research, Kochi, India; 9 Global Health Economics & Policy, Faculty of Medical Sciences, University of Kragujevac, Kragujevac, Serbia

**Keywords:** digital health, health technology assessment, decision-making, India

## Abstract

**Objectives:**

The aim of this study is to understand the path for establishing digital health technologies-health technology assessment (DHT-HTA) in India.

**Methods:**

A rapid review of HTA and DHT frameworks on PubMed (MEDLINE) and Google Scholar was conducted to identify DHT-HTA guidelines, and HTA processes in India. MS-Excel template was created with key domains for assessing DHT in resource-constrained settings based on studies and reports identified. Responses received from seventeen experts with varying expertise in DHT, HTA, clinical, and research were contacted using an online form. Following the principles of qualitative research rooted on grounded theory approach, themes and domains were derived for a framework which was again circulated through participants. Weightage for each theme was assigned based on the frequency of responses and qualifiers were used to interpret results. Inductively derived themes from these responses were clubbed together to identify macro-level systems requirements, and finally pre-requisites for setting up DHT-HTA framework was synthesized.

**Results:**

HT are commonly perceived by experts (64.7 percent participants) as a technology strictly connected to health information. Real-world data (i.e., electronic health data) are recognized as a relevant tool in support of decision-making for clinical and managerial levels. Experts identified some pre-requisites for the establishment of DHT-HTA in the country in terms of infrastructure, contextual factors, training, finance, data security, and scale-up.

**Conclusion:**

Our research not only identified the pre-requisites for the adoption of a DHT-HTA framework for India, but confirmed the need to address DHT-HTA’s acceptability among. Hospitals and health insurance providers.

## Introduction

Digital Health Technologies (DHT) refers to digital therapeutics, electronic patient monitoring systems, medical devices (diagnostic, prognostic, and/or therapeutic), wearables, biometric sensors, smartphone applications, electronic clinical tools, virtual assistance and telemedicine platforms, personal health tools, electronic medical records, web programs, artificial intelligence and machine learning programs, and other health management applications (1). Recent advancements in artificial intelligence (AI), machine learning (ML) applications, robotic devices, mobile health (mHealth), and IOT (Internet of Things) have further accelerated digital health innovations globally. In India alone, the overall market size of digital health is hovering around INR 524 billion in 2021 and is projected to cross INR 2500 billion in 2027 (CAGR @ 28 percent) (2). Strong overall trend in accelerating innovation of digital technologies applied to health care, largely led by India, is visible to a comparable extent in the Association of Southeast Asian Nations (ASEAN), European Union (EU), and East Asian countries (3). Furthermore, this is a notable moment in recent economic history that wider South Asian region is gaining momentum and a competitive sharp edge in these technologies significantly exceeding the Western European region in certain patents (4; 5).

Healthcare services could be further developed with the use of new digital healthcare technologies like robotics, AI, and mHealth, but their implementation should adhere to the same standards as traditional healthcare practices. Their effects on patients and organizations need to be made clear, and they need to be safe to use with advantages supported by evidence-based research (6). The International Network of Agencies for Health Technology Assessment (INAHTA) and Health Technology Assessment International (HTAi) defines health technology assessment (HTA) as a multidisciplinary process using explicit methodologies to determine the values these technologies can deliver to patients and their families, health systems, and society at different points in their lifecycle (7). In 2014, the World Health Assembly adopted a resolution on the use of HTA techniques to ensure Universal Health Coverage. Canada, Switzerland, and the United Kingdom are the leading countries in this field despite some challenges (8; 9). European Union developed HTA Core Model® framework under the aegis of European Network for Health Technology Assessment (EUnetHTA) (10). The core model is built to capture both broad as well as rapid-scoped HTA. EU core model encompasses the three components (i) HTA ontology – a set of 136 questions standardized assessment elements within assessment elements within a framework with nine domains (such as effectiveness, safety, organizational, economic, patient, and social aspects) that comprise all aspects potentially relevant for HTA and thus value assessment, (ii) methodological guidance and (iii) a common reporting format for all users (11). The core model is available to use for free but subject to adherence to the HTA core model license (12). National Institute for Health and Care Excellence (NICE) in the United Kingdom has also developed policies and strategies for HTA in the UK (13).

Middle-income countries, on the other hand, are still mostly involved in raising awareness, training, and skill development of HTA-related staffs, institutionalizing the concept of HTA, and allocating appropriate resources for effective and safe decision-making in their health system (14). While there are some efforts in introducing and institutionalizing HTA in low-and middle-income countries (LMICs) (e.g., Thailand, Colombia, Brazil, and India), others may be constrained by limited technical and administrative capacity, paucity of data, time, and governance structures to carry out HTA (15–18). Yet despite some obvious setback in HTA capacities, the Brazil, Russia, India, China, and South Africa (BRICS) emerging markets mostly experienced strong growth in long term towards increasing their per capita spending towards healthcare which is likely to continue well beyond 2030 (19; 20). Recently, COVID-19 has left a long-lasting impact on the healthcare infrastructure of many such countries and exposed numerous loopholes and shortfalls (21–24).

### Traditional HTA versus DHT-HTA

Traditional HTA draws upon a data modeling and evidence synthesis framework to arrive at conclusions about the clinical efficacy, clinical effectiveness, safety, cost-effectiveness, and budget impact of treatments (25). The introduction of a high number of DHTs along with the growing trend of AI and robotic solutions along with combinations of different HTAs increase the complexity of HTA methods. In order to measure added value while always keeping patient safety first, it may not be appropriate to use the traditional clinical domains of HTA (relative safety, relative clinical effectiveness) in every case of DHTs. This highlights the need to update or develop a specific methodological framework with new elements like data privacy, interoperability, usability, and different outcome categories (26). HTA frameworks to evaluate DHTs and AI applications remain limited and fragmented to date (26).

### Wide-spread use of digital technologies in health care in India

The need for HTA arose with the 12th Five Year Plan for India by Planning Commission - to consider ‘cost-effectiveness studies to frame clinical treatment guidelines’ and to assess available therapies and technologies. The division presently operating under the name Health Technology Assessment in India (HTAIn) was formerly referred to as the Medical Technology Assessment Board (MTAB). Health Technology Assessment Board Bill was passed in the Parliament in 2019 to institutionalize the structure and function of the HTAIn (27). HTAIn has completed studies pertaining to HTA on medical devices, diagnostic devices, surgical interventions, procedures, screening strategies, costing studies, HRQoL, pandemic support, and health programmes (28).

The National Institution for Transforming India (NITI Aayog), which serves as the apex public policy think tank of the Government of India, launched the National Digital Health Blueprint in July 2019 which had two key recommendations: (i) use of a unique health identity number (UHID) and (ii) the strengthening of electronic health records in the public and private health care sectors. These two recommendations are central to the basis for the future of surveillance in India, as outlined in this vision document (29). The goal of National Health Policy, 2017 is attainment of the highest level of health and well-being for ALL at ALL ages, through increasing access, improving quality, and lowering the cost of healthcare delivery. In tune with this, the Ministry of Health and Family Welfare (MoHFW) has prioritized the utilization of **digital health** to ensure effective *“service delivery”* and *“citizen empowerment”* to bring significant improvements in the public healthcare delivery. Additionally, certain eHealth initiatives using ICT aim to improve efficiency in healthcare delivery, expand healthcare access to rural regions, and deliver superior quality services at low cost (29). A few of the ongoing initiatives in Digital Health being implemented by MoHFW include Reproductive Child Healthcare (RCH), Integrated Disease Surveillance Program (IDSP), eHospital, e-Shushrut (a hospital management system), Electronic Vaccine Intelligence Network (eVIN), National Health Portal (NHP), National Identification Number (NIN), Online Registration System (ORS), Mera Aspatal (Patient Feedback System) and National Medical College Network (NMCN) (30).

On August 15, 2020, the National Digital Health Mission (NDHM) was launched to digitize India’s healthcare sector by storing all citizens’ health data in an internal database. The mission’s internet portal offers citizens standardized digital healthcare services. Digi Doctor, Health Facility Registration, Personal Health Records, and Electronic Medical Records are mission highlights. The Ayushman Bharat Digital Mission (ABDM) was launched in April 2022 to support digital health in India, which is currently in development (31). The NITI Aayog (2018) report outlines the six “pillars” of digital health in India. These include the selection of a governance entity, a health data registry, a strategy for the development of a unified health information system, a design for health insurance information systems, electronic health records (EHRs) for patients and health care providers, and the development of a health information infrastructure for the integration of the aforementioned elements (32).

Artificial intelligence (AI) has seen an exponential growth in India in the past few years. Recently, the Government of India has taken various initiatives related to AI such as the establishment of an Artificial Intelligence Task Force, the formulation of NITI Aayog’s National Strategy for Artificial Intelligence **#AIFORALL**. The path for adoption of AI-driven healthcare in India is filled with a lot of challenges such as lack of trained workforce, data privacy and security, and so forth. Applications of AI in Indian healthcare include the Indian Institute of Technology Bombay (IIT-B) incubated start-up Matra Technology which developed a mobile based AI technology for reducing pregnancy risk (33). Other examples include a non-invasive, low-cost option to early screening of breast cancer based on mapping body heat embedded with AI technique termed as ‘Niramai’, with claims to detect tumors five years earlier than mammography or clinical exams based on ‘Thermalytix’ technology (34).

With rapidly evolving digital health in low- and middle-income settings, this study aims to understand the path for establishing HTA for DHT in India.

## Methods

### Design and settings

Rapid reviews provide a systematic framework to gather and appraise the available literature about a practice or policy issue within an accelerated timeframe (36). As a first step search was conducted by single author (RNP) using search terms ‘Digital Health Technology’, and ‘Health Technology Assessment’ on PubMed(MEDLINE) for HTA and DHT frameworks. Results of the search were filtered to identify HTA frameworks, policies, programs, barriers, and facilitators of technology assessments. Additional search was conducted in Google Scholar to identify policy reports, market research, and program documents. Based on identified studies, a template was created with key domains for collecting information from participants on digital health technologies in resource-constrained settings. Search, screening, and data extraction for the rapid review were conducted by a single author (RNP).

Over 20 experts in India, with varying expertise in DHT, HTA, clinical, and research were contacted through email by the lead author (DJ) in April–May 2023 to respond to a semi-structured open-ended questions using online Google form (Supplementary Annexure 1) to understand perceptions of stakeholders on DHT-HTA in India. Experts had the option to refuse to be part of the study. These 20 experts were identified through contacts of lead author (DJ) due to prior work on research grant on AI/ML or previous trainings on HTA in India. Author team members (DJ and RNP) pursued experts over two weeks and received responses from 17 members. The response was free-listed, and coded (axial and selective codes) to identify themes and broad domains.

### Analysis and Synthesis

We followed principles of qualitative research rooted on grounded theory approach to inductively derive themes. Themes and domains were used to derive a model framework which was again circulated through participants. Weightage for each theme’s was assigned based on the frequency of responses and qualifiers were used to interpret results (Table 1). Pre-requisites for setting up a DHT-HTA framework was retrieved from two open-ended questions in Supplementary Annexure 1 (Q8 and Q29).

**Table 1.** Description of qualifiers assigned

Proportion of response	Qualifiers used	Adjectives used
<10%	<1+	Very few
10–24%	1+	Some
25–49%	2+	Approximately half
50–75%	3+	Majority
76–89%	4+	Most
> 90%	5+	Almost all

*Note:* Qualifiers were intended to select priorities based on the responses.

After consulting with our experts with specific questions (Q8 and Q29) from Supplementary Annexure 1, we embarked on a comprehensive analysis to identify the necessary macro-level system changes required to establish a DHT-HTA framework in India (as outlined in Box 1). The process involved carefully examining the recommendations provided and categorizing them into themes using a qualitative analytical framework. This allowed us to discern common threads and patterns among the recommendations, facilitating a more structured approach to understanding their implications. Subsequently, we organized these recommendations into six distinct domains, providing a systematic framework for addressing each area of concern. This approach not only helped in clarifying the key focus areas but also enabled a more nuanced understanding of the interdependencies between different aspects of the proposed framework. By employing this methodical analysis, we were able to delineate a comprehensive roadmap for implementing the necessary changes and laying the groundwork for a robust DHT-HTA framework in India.

Box 1: Pre-requisites for establishment of DHT-HTA in India

**Infrastructure**: For assessing Digital Health products in India, requires upgraded IT Infrastructures (e.g., computers, software’s, servers, uninterrupted electric and internet supply) which should comply with data safety standards (under Ayushman Bharat Scheme).

**Contextual factors**: Attempts should be made to involve primary level health centers (Govt. and Pvt sector), particularly in remote locations and rural areas, for assessing DHT products in the country.

**Training**: Health professionals must be trained on evidence-based practices to facilitate optimal usage of DHT dossiers. Dedicated health workers should be trained for conducting DHT product evaluations. Work force should be trained in collating secondary information, entering records of patients, inventories, analysis, and other aspects.

**Finance**: Moreover, ensuring the above would also mean a concerted flow of funds into health care system, rather focussed on each pillar of digital health. Hence, the health budgets of both the state and central governments will need to be increased.

**Data Security**: Establishing mechanisms to ensure data privacy and cyber security is imperative for the digitization of health care sector. Guidelines are laid under Government of India schemes such as Ayushman Bharat.

Stakeholder co-ordinations and engagement with private sectors are imperative for the success and maintenance of DHT in India.

**Scale-up**: Establish a common HTA framework under the BRICS (Brazil, Russia, India, China, and South Africa) initiative like the EUnetHTA model but customized to local needs and demands.

The following recommendations were arrived after a qualitative analysis of responses received from experts working in DHT, HTA, clinical, and health research in India:

1. Infrastructure must be upgraded and computers, proper electricity, and internet connections must be ensured in the health centers, particularly in rural areas for smooth functioning and adoption of DHT in the country.
2. Health professionals must be trained into using the technologies being made available to them to optimally use them in entering records of patients, inventories, and more. Especially training the ASHAs and ground-level health workers will be important.
3. Moreover, ensuring the above would also mean a concerted flow of funds into health care system, rather focused on each pillar of digital health. Hence, health budgets of both the state and central governments will need to be increased.
4. Establishing mechanisms to ensure data privacy and cyber security is imperative for digitization of health care sector, for example, enactment of the long pending data privacy bill in addition to incorporating a legal clause to ensure and enforce strict local storage, usage, and sunset clause on each byte of information collected by private and/or public entities.
5. Stakeholder coordination is imperative for the success and maintenance of DHT in India.
6. Attempt to establish a common HTA framework under the BRICS (Brazil, Russia, India, China, and South Africa) initiative similar to the EUnetHTA model but customized to the local needs and demands.

## Results

From the literature review, we identified country HTA guidelines for DHT for Finland, France, Germany, Korea, Spain, and the United Kingdom (37–39). Based on review of these documents over 10 domains and criteria of DHT-HTA framework was identified: 1) company information, 2) product-specific information, 3) technical stability, 4) effectiveness (clinical/medical benefit), 5) cost-effectiveness, 6) clinical safety, 7) data security and protection, 8) usability and accessibility, 9) interoperability, 10) artificial intelligence, and 11) robotics. Review of the Health Technology Assessment India (HTAIn) Web site, revealed that the HTA process in India includes: 1) the selection of topic, 2) technical partner consultation, 3) proposal development, 4) research and analysis, 5) economic evaluation, 6) appraisal of evidence, and 7) final recommendation; for considerations as part of priority-setting process (40).

From the interviews, we observed great variation in the scope, selection of methods, and level of detailing in the practice of HTA in India, across various settings. Our focus in these interviews was mostly based on HTA in LMIC settings where primary data collection methods, secondary or integrative methods exist in HTA methods. Primary methods use analysis of original data, such as clinical trials and observational studies, while integrative methods, or secondary methods, uses combination of data from existing literature sources and included analysis of primary data studies.

Description of themes that emerged from the online interviews is presented in Table 2. Majority of participants (11/17 participants) considered DHT as health information that is stored and transmitted electronically, such as patient medical records, lab results and diagnostic images, treatment histories, and other healthcare-related data. Some participants (4/17 participants) mentioned real-world data (RWD) within DHT, encompassing data collected outside of traditional clinical trials, often in real-world settings such as clinics, hospitals, and patient homes. In their view, when digitized, this data can provide valuable insights into patient outcomes, treatment effectiveness, and disease trends. Approximately half of the participants (2+) mentioned that electronic health data can serve as a powerful decision-making tool for healthcare providers and policymakers. By analyzing large datasets, healthcare professionals can make informed decisions about patient care, resource allocation, and public health strategies. Approximately half (2+) of the responders also mentioned that digital health data facilitates improved access to healthcare services by enabling telemedicine, remote consultations, and mobile health applications. The responders also mentioned the necessity for support towards more efficient healthcare delivery through electronic medical records (EMRs), automated reminders, and streamlined workflows.

**Table 2.** Description of themes for DHT-HTA framework by experts from the online survey – a qualitative synthesis from seventeen participants

Domains	Themes	Description of themes	Weightage (Qualifier)
Description of a digital health technology (DHT)	Electronic health data	Electronic medical records, medical history information, disease/health informatics, mHealth, hospital data, public health data	11 (3+)
	Real-world data in digital forms	Automated health information, Virtual devices, Sensors, robotic devices, artificial intelligence/machine learning applications, medical/diagnostic/prognostic devices, health software/Apps	4 (1+)
	Decision-making tool	Use the expertise of health specialists/clinicians, facilitate evidence generation, aid in the prevention and management of medical conditions, support decisions	8 (2+)
	Improve access and healthcare delivery	Coverage (improve access in rural settings), limited health infrastructures, online consultation, telemedicine services, app-based health interventions	7 (2+)
	Effectiveness and safety	Intrinsic safety of devices, extrinsic ability of technologies to drive safety, Resource mapping, feasibility, technology, and digital intervention uptakes, the safety of interventions, clinical and cost benefits/impacts	8 (2+)
	User engagements	End-user information, patient feedback, health expert feedback (clinician, community workers), prioritized health issues	4 (1+)
	Ethics and digital clinical safety	Data security, privacy, medical ethics	3 (1+)
	Remote monitoring	Virtual consultations, telemedicine services, 24-hour support	4 (1+)
DHT assessment framework for India	Selection of problem	Choosing a DHT problem, Evidence gap mapping, objectives of DHT, Primary and secondary data usage, Contextual factors	8 (2+)
	Selection of comparator	best available supportive care, based on clinical effectiveness, pre-post intervention, safety profiles, cost-effectiveness, ethical, governance, implementation, monitoring, acceptability, ease-of-use, exposed versus non-exposed, latent class modeling (internal gold standards)	13 (4+)
	Choosing a theoretical framework	Theories of planned behavior, Culture and value theory and flow experience theory, unified theories of acceptance and technologies	2 (1+)
	Technical components	Flexibility and adaptability, Testing of AI/ML algorithms, Use of primary and secondary data, and emerging digital technologies as they become available	3 (1+)
	Costing of technologies	Cost, Under and over-reported costs, Third party engagements, access issues	6 (2+)
	Data storage and privacy	Confidentiality, Standards, the data protection policy (misuse), data security, data hacking	8 (2+)
	Ethics, evidence, and safety	Transparency, outputs and outcome, impacts, patient safety issues, patient rights, autonomy	9 (3+)
	Regulatory frameworks	National-level body for DHTA, Devices for regulating digital diagnosis, prevention, monitoring, supporting life, use of software and apps in health	4 (1+)
	Collaboration and end-user participation	Developers, end-user, policymakers, clinicians, and advocacy groups, accessibility, usability, technical reliability, accuracy, and integration of digital health technologies	14 (4+)
Challenges for health providers in adopting digital technologies	Time constraints	Lack of sufficient time, resistance to change management, redundancy	7 (2+)
	Resource constraints	Financial capability, trained manpower, infrastructure costs, electricity, internet, skill training, repairs	7 (2+)
	Training and skill building	Technical know-how, training and support, a regulatory framework that addresses data privacy and security concerns	5 (2+)
	Rapid changes in technologies	Rapid changes in technologies (e.g., constantly changing point of care)	4 (1+)
	Acceptance from patients	Lack of knowledge, mistrust, superstition, cultural appropriateness, political will, and capacity to use	4 (1+)
Information from the producer of technology	Product development and testing information	Situation and needs for new technologies, technical information (blueprints), components used, testing information or trials, social and health impacts, development process	11 (3+)
	Regulatory applications and approvals	Intellectual property, compliance to standards during product development, company history, product costing and financial information, quality, and safety of components used	8 (2+)
	The business model of the company	Competitor information, the business model of product/company, revenue model, manpower requirements, company headquarters, director info, shareholding pattern, tax returns, ownership of the company, healthcare industry partners	14 (4+)
	Data management and quality assurance protocols (SOPs)	Capability and skillsets of end-users, data standards, data storage policy, the process of storage and duration of storage (how long in the case of images), data collection and management protocol (SOPs)	12 (3+)
	Data security protocol	Server location, type, and security standards Required technical rigor, Consistency, and privacy maintenance measures	13 (4+)
Framework for classifying risk	Risk classification of digital products	Need to classify digital health interventions/products based on their potential risk to patients/end-users	15 (4+)
	End-user feedbacks	User satisfaction and experience, to improve quality and safety, evaluate patient satisfaction levels, best practices on electronic health records	12 (3+)
	Automated real-world data (IOT based or sensors)	Monitoring vital signs, tracking physical activity, assessing sleep patterns, managing chronic conditions, Identifying environmental hazards, intensive care units, hospital wards, GP offices, lab equipment, dental practices, and in-home care products	4 (1+)
Assessment Framework for clinical effectiveness	Clinical benefits	Patient outcomes, Life years saved (QALY and DAKY), accuracy and precision, time saved and ease of processes (monotonous processes that could be quickened), early diagnosis, end-user acceptability, single-arm studies or real-world data, continuous data on individuals and populations, clinical pathways and practice guidelines	14 (4+)
	Cost benefits	**Direct:** Labour/HR cost, Production cost, Material cost (components), Manufacturing & supply cost, development and implementation costs, maintenance and support costs, training costs, operational costs, direct and indirect medical costs, out of pocket expenses, capital cost and noncapital cost, health expenditure, cost of storage, cost of device and delivery, transport cost, marketing cost, uptake cost, and maintenance cost, packaging cost, and R&D cost	17 (5+)
		**Indirect:** Loss of productivity, absenteeism, caregiver costs, loss of income, accessibility cost, equity cost, annual maintenance, insurance, cost of patent (IP rights), technology failures, maintaining web portals/apps, time and travel costs, intangible costs, environmental costs, administration and support, office and equipment rental costs, and investment write-offs	14 (4+)
	Adverse events	Improper use, wrong interpretation of the results (human errors), risk assessments, evaluate mitigation strategies, materio-vigilance programs, deep learning AI enables the identification of AE patterns that may not be easily recognizable by humans	9 (3+)
	Biases in Digi HTA	Selection bias, expertise of users, environmental issues (temperature, storage conditions, power source, etc.), conformity bias, gender bias, recall bias, reporting bias, algorithm bias	10 (3+)
	Technology readiness levels (TRL)	Ensure safety, assess the feasibility, effectiveness, and impact of digital health interventions	6 (2+)
	Contextual information	Adaptability in the Indian context, Cultural, language, and social aspects, accessibility to rural and remote populations, ease of use, availability, and affordability	12 (3+)

*Note:* Types of digital health technologies considered are digital therapeutics, patient monitoring/remote monitoring, medical devices (diagnostic and therapeutic), wearables (assisted living), biometric sensors, smartphone-based applications, clinical/diagnostic tools, virtual assistance and telemedicine, personal health, electronic health records, web programs, and disease/health management applications.

A DHT assessment framework for India was suggested by participants. Approximately half of participants (8/17) quoted that, identifying a specific healthcare challenge or opportunity that DHT can address is the first step. This could involve improving patient outcomes, enhancing access to care, reducing healthcare costs, or streamlining administrative processes. Most participants (13/17) suggested selecting an appropriate comparator or control group is crucial in a DHT framework. This could involve comparing outcomes with standard care practices, alternative interventions, or historical data.

Grounding the digital health initiative within a theoretical framework can provide a structured approach to understanding and addressing the underlying mechanisms of change. Some (2/17) suggested a common framework that include behavioral theories (e.g., Health Belief Model, Social Cognitive Theory) and implementation science frameworks (e.g., RE-AIM, Theoretical Domains Framework) is essential for DHT. Others suggested to consider the technical components required to implement a digital health solution, such as hardware (e.g., mobile devices, sensors), software (e.g., mobile apps, data analytics platforms), and networking infrastructure (e.g., cloud services, internet connectivity).

## Discussion

Ours was the first study in a LMIC setting such as India for a DHT-HTA framework. We used responses from seventeen experts working with digital health, clinical services, health research, and/or HTA in the country, and identified some pre-requisites and recommendations for DHT-HTA framework for India.

According to the NDHM blueprint, India has demonstrated its commitment to using digital health as a health system-strengthening intervention. Low public health spending, an unprepared infrastructure, and a lack of a minimal standard for gathering and maintaining health records are just a few of the problems that the authors pointed out (41). In 2021, India had the second-highest proportion of cyberattacks against the healthcare industry (7.7 percent of all attacks), which amounted to more than 7.1 million records (35). The proposed allocation for ABDM for 2023–24 has increased by 144 percent from revised estimates of 2022–23 to Rs 34.1 million (42). However, the efficacy of it is still to be seen. These aspects are of significant worry regarding the rollout of DHT in India.

It is possible to assess therapy efficacy and patient safety results by using electronic health records data. Through longitudinal monitoring of patient reactions to various interventions, healthcare providers can determine the most effective therapies while reducing side effects. Using digital tools and platforms to involve patients in their own healthcare is becoming more and more crucial. Patients may monitor their progress, manage their health more efficiently, and interact with healthcare providers with the use of electronic health data. Making sure that healthcare data is securely stored and used ethically is crucial as the industry grows more digitalized. This entails adhering to legal standards, preserving data integrity, and safeguarding patient privacy. Patients with chronic diseases, the elderly, and those receiving post-operative care can all be monitored remotely thanks to electronic health data. Healthcare practitioners can take early action to avoid issues and enhance results by continuously gathering and evaluating patient data remotely.

Time and budget constraints, patient acceptability, talent development and training, and quick technological advancements are all important considerations when introducing DHTs. The process of developing a digital health solution includes planning, constructing, and optimizing it. Government agency regulatory clearances may be necessary, depending on the target market and the nature of the digital health product. To make sure the product is reliable, usable, and functional, testing is essential. A variety of testing techniques, such as unit, integration, user acceptability, and performance testing, may be used in this. Traceability and regulatory compliance depend on the product development process’s documentation, which includes design documents, test plans, requirements, and results. DHT’s business model ought to specify how it makes money and provides value to clients. Common business models in the area of digital health include pay-per-use schemes, license contracts, subscription-based services, and alliances with payers or providers of healthcare. Procedures for gathering, storing, processing, and analyzing data are established by data management protocols, which also guarantee data availability, confidentiality, and integrity. Data backup techniques, data security measures, and data governance principles are a few examples of this. Standard Operating Procedures (SOPs), which are frequently used to establish quality assurance methods, set forth the steps involved in guaranteeing the dependability and caliber of goods and services.

Digital products, especially those in the healthcare domain, are subject to various risks that need to be identified and managed. These risks can include data breaches, software failures, regulatory non-compliance, and patient safety concerns. Risk classification involves categorizing these risks based on their severity and likelihood of occurrence. End-user feedback is essential for evaluating the usability, effectiveness, and satisfaction with digital products. This feedback can come from patients, healthcare providers, administrators, or other stakeholders involved in the use of the product. Automated real-world data refers to data collected from sensors, Internet of Things (IoT) devices, wearables, and other connected technologies in real-world settings. Further, digital health technology framework should focus on clinical benefits, economic benefits, unintended consequences, and document the technology readiness levels. By understanding and addressing these factors, stakeholders can maximize the clinical and cost benefits of digital health technologies while mitigating risks, biases, and contextual challenges.

Currently, the HTAIn Secretariat has established liaison with various national and state agencies such as the Ministry of Health Family Welfare, NITI Aayog, National Health Authority, National Pharmaceutical Pricing Authority, Central Drug Standards Control Organization, and various Departments of Health in various states (18). With multiple players at national and state levels, our results to be applied at national, state, district, and hospital levels requires a clear procedure in place for HTA process in decision-making in India, and hence would be difficult to incorporate into the healthcare decision-making process in the country. Such situations exist in other countries as well; for example, in Finland, there is currently no formal process for integrating Digi-HTA recommendations into the healthcare decision-making processes (37). However, increasing the use of DHT-HTA in India might be possible if assessments for some DHT products were included in both public and private health insurance in the nation as part of reimbursement process, as is the case in Germany (43).

Another aspect to consider in India as part of DHT-HTA framework is joint HTA procedures at district or state level for implementing HTA. For example; according to the Finnish Health Care Act from 2010, hospital districts must agree on the guiding principles for implementing new medical techniques and coordinating publicly-funded delivery of highly specialized medical care within their catchment area. As a result, university hospitals have established joint HTA procedures (44).

Effectiveness, costs, and safety have historically been the three main considerations in HTA recommendations; however, our online survey revealed that along these aspects data security, protection, and end-user engagement must also be fully taken into account for DHT-HTA in India.

According to the responses to our online survey, the term DHT can refer to a wide range of products, such as information collection techniques (like electronic medical records), automated healthcare solutions (like health apps, AI/ML for disease prognosis, etc.), or use as a disease support system for doctors managing diseases. DHTs are heterogeneous, therefore the context in which they are used will have an impact on how effective they are (e.g., tribal populations). While some DHTs are utilized by healthcare professionals, others are directed at the general public, necessitating that the DHTs be user-friendly and available to all users. Our survey demonstrates the necessity of including technology readiness levels (TRL) and contextual data in DHT-HTA in India.

The use of DHT for enhancing access and healthcare delivery to increase coverage in rural areas, places with poor health infrastructure, and app-based health interventions was also noted in the responses to our online poll. The World Health Organisation (WHO) is in favor of using telemedicine to provide medical treatment in areas with insufficient medical resources (38). After virtual care has been adopted, HTAs should consider issues like the right medical conditions for virtual care, training, billing, patient and clinician perspectives and experiences, and equity of access (39). For HTA organizations, it is critical to recognize new and developing virtual care technologies, investigate early and different types of evidence, evaluate the possible influence on the healthcare system, and investigate operational factors (45).

Half of the studies of a systematic evaluation of 178 DHT treatments in high- to middle-income countries included all dimensions, with the exception of the effectiveness and ethical analysis domains (46). For primary DHT research, the effectiveness assessments must be carried out via high-quality clinical trials and ethical reporting; failure to do so could result in health services making an unfavorable investment decision. One such instance in the country is the Bangalore-based company “Niramai” use of breast thermography/infrared imaging of the breast as a screening tool for the initial detection of breast cancer (41). The business and other medical professionals submitted an article in JCO Global Oncology titled “Observational research to evaluate the clinical efficacy of Thermalytix for identifying breast cancer in symptomatic and asymptomatic patients” (47). By contrasting the CADx output with the final diagnosis reached using conventional screening modalities, this study examined the sensitivity and specificity of Thermalytix, an artificial intelligence-based computer-aided diagnostics (CADx) engine, to detect breast cancer (47). All of the authors disclosed their potential conflicts of interest and were either paid by or employed by the company. ML models were retrained using 102 participants from the 587 female participants. A total of 343 women over the age of 40 and 127 women under the age of 40 were recruited for the prospective study. Neither a reference standard nor a two-year follow-up were used. A statement stating that “The Society of Breast Imaging India does not extend its support for using breast thermography/infrared imaging of the breast as a screening tool for the primary detection of breast cancer or as an adjunctive diagnostic tool for detection of breast cancer” was published on the Web site of the Society of Breast Imaging India as a result of these facts (48). DHT developers and implementors would need a new strategy and direction as part of the DHT-HTA framework for India in order to produce research data on the efficacy of their present and upcoming products. For example, in Germany and the UK, companies are instructed on the type of proof needed to prove the efficacy of their products in order to get reimbursed for their goods (43;49).

Early health technology assessment (early HTA) has served as a guide for the innovation development process in the medical technology development industry (50). The use of a DHT-HTA framework for India could assist both new and establishe+d DHT enterprises in understanding the important factors to take into account for DHT prior to releasing their products on the market. We believe that through these online responses were gathered from experts based in India, the results generated could be applicable to other developed and developing countries where DHT are being implemented and progressing.

## Limitations

Along with rapid review of published literature, our study was conducted using an online survey, and this may have an impact on the qualitative analysis of steering the respondent’s opinions in a particular direction. Notwithstanding these limitations, we feel that this study could lead to a potential debate on the topic of DHT-HTA in India, and needs further research and analysis so as the facilitate decision-making by users, hospitals, and public health systems in the country. Additional research with more samples or more precise study protocols is needed to generalize the findings of this study.

## Conclusions

The field of DHT covers a wide range of technology solutions such as digital therapeutics, electronic patient monitoring systems, medical devices, wearables, biometric sensors, smartphone applications, electronic clinical tools, virtual assistance and telemedicine platforms, personal health tools, electronic medical records, web programs, AI/ML programs, and other health management applications. Responses from our online survey in the country indicate the need for establishing a DHT-HTA framework for India for assessing DHT products in the country, and the biggest improvement is needed for the usability and accessibility domain to make it comprehensive for all kinds of DHTs. Besides political will and funds, acceptance of the DHT-HTA by health insurance agencies and hospitals would be an important factor in ensuring the success of DHT-HTA implementation in India.

## Supporting information

John et al. supplementary material John et al. supplementary material

## Supplementary material

The supplementary material for this article can be found at http://doi.org/10.1017/S0266462324000345.
