# Supplementary material for: Health technology assessment for digital health Technologies in India: a framework for action
Source: Int J Technol Assess Health Care. 2024 Nov 4;40(1):e70. doi: 10.1017/S0266462324000345 (PMC11703630; doi:10.1017/S0266462324000345)
Supplement: John et al. supplementary material [file S0266462324000345sup001.docx]

**Annexure I**: **Scoping the Path for Health Technology Assessments (HTA) and Outcome Research in digital health innovations in India**

Health technology assessment (HTA) refers to the process of assessing the impacts of a health intervention - in terms of clinical effectiveness, economic benefits, and social and ethical influence. With the current pro-business start-up ecosystem in India, many new digital technologies are rapidly released in the market for patient care (e.g., diagnostics, therapeutics, patient monitoring systems, etc.). Some of them use inbuilt algorithms (ML/AI-enabled) that may show contextual variation in their effectiveness. Few technologies may be cost-effective but fail in the market due to poor social acceptance. Situation is exacerbated with complex processes in real-world data and evidence synthesis.

In this context, we constituted a Delphi panel to list recommendations that can pave the path to frame policies on digital health technology assessment in India.

Opinion from responders will be kept confidential and deidentified information will be summarized in tables. The information collated will be disseminated in a manuscript. All participants will be duly acknowledged.

1. Name of the respondent
2. Your affiliation (designation & institution)
3. Year's of experience in health technology assessment (HTA) or clinical and/or outcomes research (economic evaluations, epidemiology, systematic reviews etc.)
4. 0 to <3 years
5. 3 to <10 years
6. 10 to <15 years
7. More than 15 years

***Digital health & HTA***

*It refers to the use of digital health information for improving patient care. Innovations in mobile technologies, artificial intelligence (AI) and robotics have great potential to improve healthcare delivery, however, it requires scrutiny of their impacts on patients. While health providers are looking for which digital tools are appropriate for their use (benefits in daily work), producers/companies are looking for information that facilitates regulatory approvals, and policy-makers are looking for evidence to support decisions. A new analytical framework has to be developed that will facilitate digital health technology assessment in the Indian context*.

1. How do you define an evidence based digital health technology ? (as per your perception)
2. According to you, what core domains should be considered for digital HTA in the Indian context?
3. Suggest a theoretical / analytical framework for developing digital HTA guidelines in India ?
4. What are the key concerns regarding a digital health technology, from a patient perspective ? (e.g., data security, privacy, large volume data)
5. What are the common challenges faced by a healthcare provider in India in adapting to a new digital health technology?
6. Is there a need to ensure patient / end user participation in the digital technology development process?
7. Yes
8. No
9. May be
10. What company information's are important in DHT-HTA process?
11. Can it be mandated to collect information on product testing methods used by the company ?
12. Yes
13. No
14. May be
15. Can it be mandated to collect company's business model during HTA process?
16. Yes
17. No
18. May be
19. Whether data management, analysis plan, and quality assurance processes by the company to be included in the digital HTA processes?
20. Yes
21. No
22. May be
23. How end user feedback's collected by the company can be used in the digital HTA process?
24. Is there a need to classify digital health intervention / product based on their potential risk to patients/ end-user (e.g., medical device risk classification system, ICD codes etc.) ? (specific to mhealth, AI or robotics)
25. How to use information / data from sensors?
26. How to assess clinical benefits of a digital health technology ?
27. How to assess unintended consequences of a digital health technology?  (e.g., adverse events)
28. Is there a need for experimental design in digital health interventions ?
29. Yes
30. No
31. May be
32. If yes, how should a comparator be selected in digital health?
33. What possible biases should be considered?
34. How to use information on technology readiness level (TRL) in the digital HTA process? (<https://www.birac.nic.in/desc_new.php?id=443>)
35. Whether post-market surveillance of the digital health product to be included in the decision making process?
36. Yes
37. No
38. How to use safety information of digital health (including data protection policies) ?
39. What all direct costing variables to be included in the cost-effectiveness of digital HTA in Indian context ?
40. What indirect costing variable to be considered ?
41. Is it important to check how companies decided on the product cost / MRP (maximum retail price) during HTA process?
42. Yes
43. No
44. May be
45. What clinical benefits to be considered when assessing cost-effectiveness of digital health in India ?
46. Is there any other recommendations important for digital HTA in Indian context?
47. Any feedback for us
